# “Rolled-upness”: phenotyping leaf rolling in cereals using computer vision and functional data analysis approaches

**DOI:** 10.1186/s13007-015-0095-1

**Published:** 2015-11-14

**Authors:** X. R. R. Sirault, A. G. Condon, J. T. Wood, G. D. Farquhar, G. J. Rebetzke

**Affiliations:** Research School of Biology, Australian National University, GPO Box 475, Canberra, ACT 2601 Australia; CSIRO Agriculture, GPO Box 1600, Canberra, ACT 2601 Australia; Fenner School of Environment and Society, Australian National University, Canberra, ACT 0200 Australia

**Keywords:** Leaf water potential, Polyethylene glycol (PEG), Mean curvature, Convex hull, Spline analysis, Digital phenotyping

## Abstract

**Background:**

The flag leaf of a wheat (*Triticum aestivum* L.) plant rolls up into a cylinder in response to drought conditions and then unrolls when leaf water relations improve. This is a desirable trait for extending leaf area duration and improving grain size particularly under drought. But how do we quantify this phenotype so that different varieties of wheat or different treatments can be compared objectively since this phenotype can easily be confounded with inter-genotypic differences in root-water uptake and/or transpiration at the leaf level if using traditional methods?

**Results:**

We present a new method to objectively test a range of lines/varieties/treatments for their propensity of leaves to roll. We have designed a repeatable protocol and defined an objective measure of leaf curvature called “rolled-upness” which minimises confounding factors in the assessment of leaf rolling in grass species. We induced leaf rolling by immersing leaf strips in an osmoticum of known osmotic pressure. Using micro-photographs of individual leaf cross-sections at equilibrium in the osmoticum, two approaches were used to quantify leaf rolling. The first was to use some properties of the convex hull of the leaf cross-section. The second was to use cubic smoothing splines to approximate the transverse leaf shape mathematically and then use a statistic derived from the splines for comparison. Both approaches resulted in objective measurements that could differentiate clearly between breeding lines and varieties contrasting genetically in their propensity for leaf rolling under water stress. The spline approach distinguished between upward and downward curvature and allowed detailed properties of the rolling to be examined, such as the position on the strip where maximum curvature occurs.

**Conclusions:**

A method applying smoothing splines to skeletonised images of transverse wheat leaf sections enabled objective measurements of inter-genotypic variation for hydronastic leaf rolling in wheat. Mean-curvature of the leaf cross-section was the measure selected to discriminate between genotypes, as it was straightforward to calculate and easily construed. The method has broad applicability and provides an avenue to genetically dissect the trait in cereals.

**Electronic supplementary material:**

The online version of this article (doi:10.1186/s13007-015-0095-1) contains supplementary material, which is available to authorized users.

## Background

The leaves of many important cereal crops including sorghum, maize, rice and wheat roll (transverse rolling of the leaf lamina along the mid axis) in response to drought conditions then unroll to continue photosynthesis when water is available. This is a trait identified as potentially important in rainfed conditions [1], particularly if late rains occur during grain-filling. It may lead to a delay in the onset of leaf senescence (longer maintenance of leaf area) and thus lead to greater water-use efficiency. The trait is mainly expressed in the flag leaf [2], which is one of the main organs contributing to grain dry weight [3, 4], a major component of final harvested yield and an important parameter in grain quality.

Significant genotypic variation for leaf-rolling has been reported within wheat germplasm [2]: leaves of some wheat varieties roll after only mild water stress whereas leaves of other varieties roll only when severely water-stressed. For use in breeding, a measure is required to quantify rolling so that different genotypes and/or different treatments can be compared objectively. Apart from the obvious differences in leaf rolling arising between genotypes from variation due to evaporative demand and/or spatial heterogeneity in soil water content in the field, this phenotype is easily confounded with inter-genotypic differences in root-water uptake and/or transpiration at the leaf level. To understand the leaf rolling phenotype one must be sure of the intrinsic value of the tested lines.

The degree of rolling in the flag leaf is often quantified by using a visual discrete scale of schematic transversal shape (leaf rolling score from 1 ≡ flat to 5 ≡ tightly rolled) [5], or by using a rolling index defined as the ratio of rolled leaf width to unrolled leaf width [6]. The choice between leaf-rolling score and leaf rolling indices is a trade-off between a rapid but qualitative measure if using leaf-rolling scores and a slow but quantitative measure if using rolling indices. Applying a visual score on different plant species requires different transversal references [7] and raises the problem of the definition of leaf rolling. This is particularly relevant since leaf rolling tends not to be homogeneous along the midrib, i.e. the tip is usually more rolled than the middle of the leaf. In addition, visual ratings of different “scorers” cannot be compared or related adequately to each other [8] so there is a need for a repeatable protocol for screening leaf rolling genotypes. Using rolling indices address some of these issues because they are calculated ratios but they are not logistically practical under field conditions and are highly confounded with environmental factors when used in situ.

**Fig. 1:**
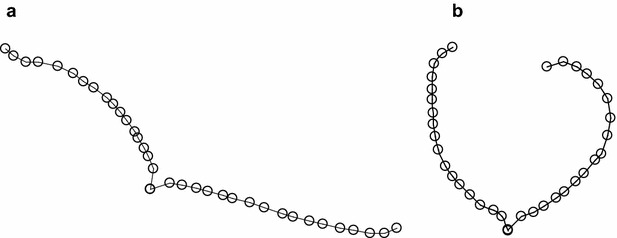
*Digitised curve*s for two strips from the same leaf: **a** before dehydration and **b** after dehydration

A quantitative method, which would minimise all confounding factors, i.e. assess all lines in the same conditions, is therefore essential for comparing genotypic differences for extent of rolling. There is thus a need to develop: (1) a repeatable protocol that will minimise those factors that could confound the leaf-rolling phenotype; and (2) develop an objective index that would discriminate genotypes for leaf rolling. Our strategy aimed at examining the changes occurring in leaf transverse shapes when imposing a certain level of water stress to the leaf since leaves readily change transverse shape when subjected to dehydration (Fig. 1). For a reliable protocol the challenge was to impose conditions that allowed the change in shape to be measured in a reproducible manner. The experiments reported here tested whether such repeatable conditions could be achieved by immersing narrow, transverse leaf sections into solutions of PEG of differing concentrations allowing the leaf strips to reach thermal and osmotic equilibrium with the solution before being micro-photographed. The second aspect of the study was to define a continuous index that was an appropriate measure of the degree of rolling for the individual strips and that could be used as a response variable in the analysis of the data. As such, this paper considers a situation in which the response of the leaf to the osmotic solution is described by a continuous curve and our interest is in the property of this curve, the degree of “rolled-upness”, which is easy to describe in general terms, but is less easy to capture as a precisely defined property [9, 10]. In an attempt to achieve this, the micro-photographs of the individual leaf sections were processed and the transverse shapes described mathematically. Two indices were calculated: (1) a mean curvature index derived from the curvature profiles of the leaf strips and (2) the maximum diameter of the convex hull expressed as logarithm of the ratio of leaf strip length to maximum diameter of the convex hull.

## Results

**Fig. 2:**
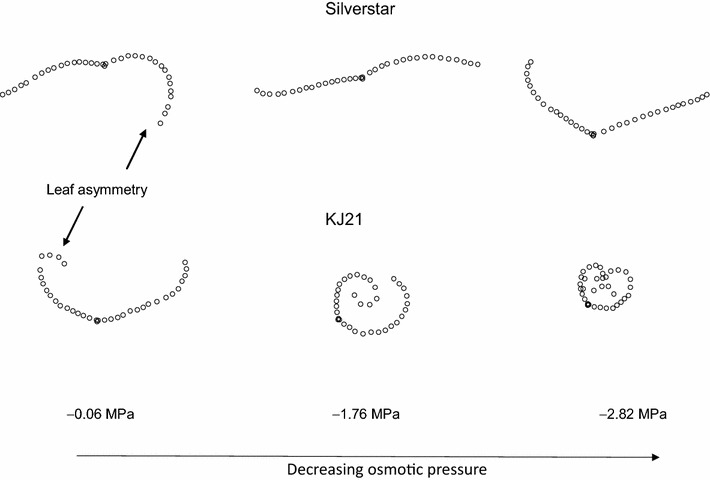
Contrasting responses to dehydration in Silverstar and KJ21 at osmotic potentials of −0.06, −1.76 and −2.82 MPa

Figure 2 represents the transverse shape of wheat line KJ21 and *cv.* Silverstar at equilibrium in three polyethylene glycol [PEG] 3350 solutions with respective osmotic pressures of −0.06, −1.76, and −2.82 MPa. At equilibrium, the leaf water potential of the strip is equal to the osmotic potential of the PEG solution. The transverse shape changed with increasing PEG concentration, i.e. decreasing osmotic potential. The complete series of transverse shapes for those two genotypes over the seven osmotic solutions is presented in Additional files 1 and 2. It is worth noting that at −0.06 MPa (near full turgor), the transverse shapes for both *cv.* Silverstar and line KJ21 presented a contrast in concavity: a negative concavity or “outward” rolling for *cv.* Silverstar (oriented towards the abaxial surface) and a positive concavity or “inward” rolling for line KJ21 (oriented towards the adaxial surface). As the leaf water potential decreases, the concavity of *cv.* Silverstar reversed progressively towards the adaxial surface. This had become particularly obvious at very low osmotic potentials (−2.82 MPa) (Fig. 2). At intermediate osmotic potential (−1.76 MPa), the transverse shape was almost flat. However, for line KJ21, which was slightly rolled inwards at full turgor, rolling increased almost immediately to finish highly rolled at −2.82 MPa. The process was reversible for all lines tested however hysteresis was not quantified.

### Development of a data analysis framework for phenotyping leaf rolling

To measure the degree of rolling for the individual strips we considered two approaches as follows:

#### Convex hull

**Fig. 3:**
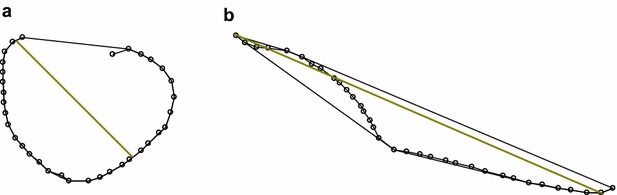
Convex hull and longest diagonal of the convex hull (in *yellow*) for the strips in Fig. 1, considering the whole cross-section excluding the midrib

One possible approach was to consider the convex hull of the transverse shapes, i.e. the smallest convex set of points that includes the cross section (Fig. 3). Highly rolled strips have a compact convex hull, with a small maximum diameter relative to an unrolled strip. Note that parts of the strip itself may lie outside the convex hull. The ratio of the maximum diameter of the convex hull to the length of the strip represented the degree of “rolled-upness”. We used a logarithmic form of this ratio to scale the standard errors.

#### Smoothing spline functions

**Fig. 4:**
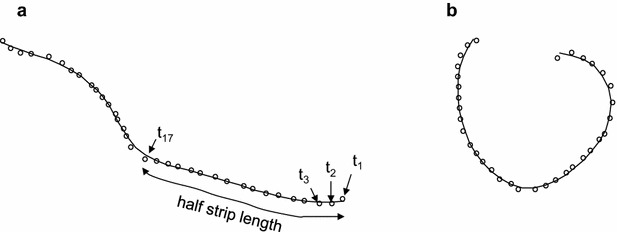
Smoothing spline functions for the leaf cross section in Fig. 1; *digitised curves* for two strips from the same leaf, **a** before dehydration and **b** after dehydration. *t* is a variable which indexes position along the *curve*

An alternative approach consisted of fitting smoothing spline functions to the leaf cross-sections. For this method, one starts by defining a variable, *t*, which indexes the position of digitised points along the cross-section (Fig. 4: *t*_1_–*t*_17_). For convenience *t* can be chosen to have integer values at the digitised points, which were chosen to be roughly equally-spaced. We then represented the cross-section by fitting smooth functions of this indexing variable to the observed vectors $\varvec{x}$ and $\varvec{y}$, describing the coordinates of the digitised points. Some function of the curve (i.e. descriptors) such as the average curvature or the maximum curvature can then be estimated for each strip. To smooth the curve and remove random variation in the sampling of the points used to describe the transverse shape, we replaced the vectors of observations, $\varvec{x}$ and $\varvec{y}$, by $\varvec{x}_{s} = S\varvec{x}$ and $\varvec{y}_{s} = S\varvec{y}$, where *S* is a smoothing matrix. This smoothing operation is invariant under rotation of the coordinates since $ax_{s} + by_{s} = S(ax + by)$. Any smoothing matrix could be used but we chose to fit cubic smoothing splines to the data because this enabled us to estimate derivatives at all points on the curve. Since we were interested in capturing the overall shape of the strip in response to the imposed water potential without being too sensitive to local variation in curvature along the strip width, cubic smoothing splines were an ideal fitting technique to achieved the desired outcomes, in addition to being easily implemented. Curvature is defined as the amount by which a geometric object deviates from being flat. In other words, curvature at a given point P has a magnitude equal to the reciprocal of the radius of the osculating circle at point P, i.e. the best circle that approximates the curve at point P [16]. The vector pointing in the direction of that circle’s centre indicates the sign of the curvature; we treated curvature as positive when the centre of curvature was on the top side of the leaf (adaxial surface), and negative when it was on the bottom side (abaxial surface).

From these splines first and second derivatives, $\dot{x}_{s}$ and $\dot{y}_{s}$, $\textit{\"{x}}_{s}$ and $\textit{\"{y}}_{s}$ of *x* and *y* with respect to *t* at any point along the strip were calculated. The curvature, *κ*, at a point was estimated by, $\kappa = \frac{{\textit{\"{y}}_{s} \dot{x}_{s} - \textit{\"{x}}_{s} \dot{y}_{s} }}{{\dot{s}^{3} }}$, where $\dot{s} = \surd (\dot{x}_{s}^{2} + \dot{y}_{s}^{2} )$.

The length of the strip was estimated by integrating $\dot{s}$.

With this framework in place, several choices had to be made for analysing the data. These included the degree of smoothing to use and the points at which the curvature was to be estimated. We also had to decide what function we were going to use in the interpretation of the data (e.g. mean curvature over the whole cross-section, or position of the maximum curvature for each strip), and, since not all leaves had the same width, what standardisation, if any, was to be used.

### Effect of smoothing and interpolation on inferences

**Fig. 5:**
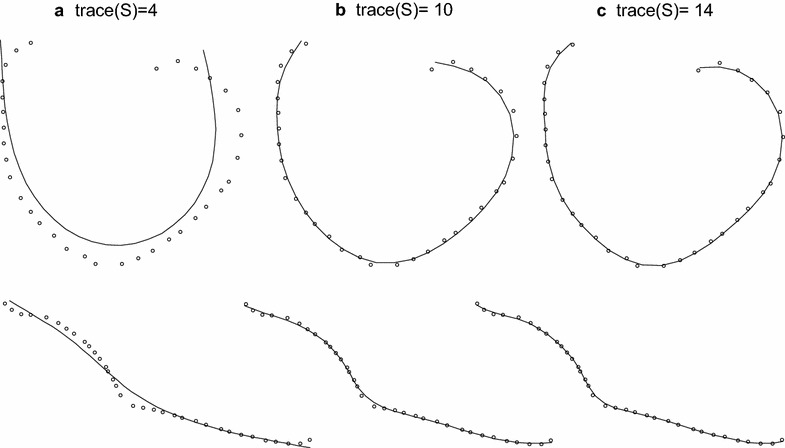
Different degrees of smoothing for the leaf cross section in Fig. 1b: **a** trace(S) = 4, **b** trace(S) = 10 and **c** trace(S) = 14

A range of levels of smoothing, defined as trace(S), was used, and we estimated the derivatives of *x* and *y* at the observation points, and at equally-spaced points between them as defined by the value of *t* (using interpolation). Figure 5 shows the results of using three different degrees of smoothing for the leaf shown in Fig. 1b. From these data it is clear that choosing trace(S) = 4 over-smooths but there is little difference in smoothing between trace(S) = 10 and trace(S) = 14. These traces correspond to fairly light smoothing. Note that the higher the trace(S), the lesser the smoothing of the spline; no smoothing would force the spline to go through all the points thus giving a perfect fit. The effect of the choice of interval for interpolation was negligible (data not shown), but over-smoothing, i.e. smaller traces, can lead to loss of information as would be indicated by reduced variance ratio. The level of smoothing obtained with trace(S) = 10 was considered to be optimal for capturing the overall shape of the strip through the use of spline functions without being too sensitive to local variation in curvature along the strip width.

### Repeatability of curvature measurements

**Fig. 6:**
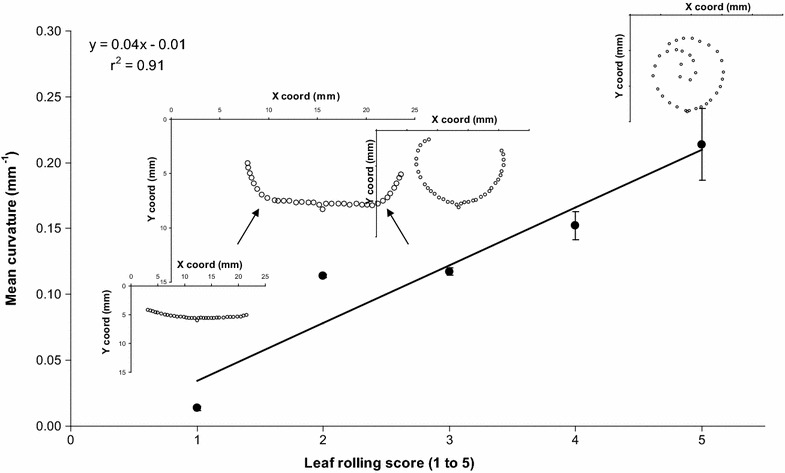
Relationship between mean curvature and leaf-rolling score as defined in O’Toole and Cruz (1979). Standard *error bars* are shown

To assess the repeatability of manually processing a digitised transverse section, the five transverse shapes comprising the visual reference scale for leaf rolling published in O’Toole and Cruz [5] were digitised using a flatbed scanner and processed as done for a normal leaf strip. Each individual transverse shape was processed five times to obtain estimates of repeatability and error associated with the method. The relationship between leaf-rolling score and mean curvature is presented in Fig. 6. Mean curvature was positively correlated (r = 0.95, *P* < 0.01) with leaf-rolling score. The size of the error bars indicated that sampling errors due to manual processing were small and repeatability was high. Therefore, quantifying leaf rolling using the spline-fitting approach, in particular by estimating mean curvature, reliably identified variation in leaf rolling as per earlier methods [5].

### Use of developed indices to characterize genotypic differences

**Fig. 7:**
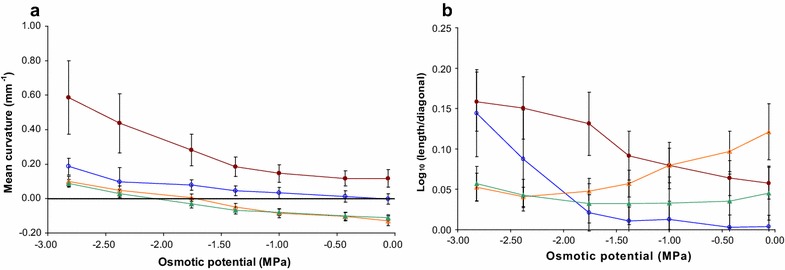
**a** Relationship between mean curvature and osmotic potential of bathing solution for rolling group genotypes B403D (*circle*) and KJ21 (*filled circle*) and non-rolling group genotypes Silverstar (*triangle*) and Diamondbird (*filled triangle*). Standard *error bars* are shown; **b** relationship between logarithm of the length divided by greatest diameter of the convex hull and osmotic potential in B403D (*circle*), KJ21 (*filled circle*), Silverstar (*triangle*), and Diamondbird (*filled triangle*). Standard *error bars* are shown

For clarity, only four of the lines used in this study are plotted in Fig. 7, which shows the relationship between leaf water potential and mean curvature (Fig. 7a) and leaf water potential and the logarithmic index (Fig. 7b). Splines were fitted with trace(S) = 10 and an interpolation interval of 0.25. For all genotypes, mean curvature increased in a monotonic way with decreasing leaf water potential. At full turgor, mean curvature for breeding line B403D was close to zero while line KJ21 had a positive mean curvature. Both genotypes were very responsive to decreasing osmotic potential. Conversely, *cvs* Silverstar and Diamondbird had a transverse shape described on average by negative curvature at full turgor, which reflected the revolute transverse shape of these cultivars (Additional file 5). Both of these varieties reached a mean curvature of zero, i.e. a flat transverse shape, when their tissue water potentials were between −1.8 and −2.1 MPa.

The same conclusions were achieved when using all genotypes (Table 1). Mean curvature at full turgor was actually negative in the non-rolling group (k = −0.137 mm^−1^) while it was positive in the rolling group (k = 0.120 mm^−1^). Interestingly, these estimates indicate that leaf rolling could be assessed under well-watered conditions. Nonetheless, differences between groups became more discernible at lower osmotic potential or higher PEG concentration; e.g. mean curvatures at PEG = 0.45 g.g^−1^ (−2.82 MPa) were 0.022 and 0.452 mm^−1^ for the low-roller and high-roller groups, respectively.

**Table 1:** Means of two measures of rolling: (1) the logarithm of the ratio of leaf strip length divided by the greatest diameter of the convex hull and (2) the mean curvature among rolling groups

	Log (length/diagonal)			Mean curvature (mm^−1^)		
	Osmotic potential of bathing solution			Osmotic potential of bathing solution		
	−0.06 MPa	−1.38 MPa	−2.82 MPa	−0.06 MPa	−1.38 MPa	−2.82 MPa
Low rollers	0.129	0.049	0.091	−0.137	−0.078	0.022
High rollers	0.163	0.275	0.683	0.120	0.159	0.452

It is also interesting to note that errors on mean curvature estimates increased at lower osmotic pressures. This indicated that variance was not homogeneous across osmotic potentials and thus caution needs to be exercised when statistically comparing mean curvature across osmotic potentials. However, increases in standard errors could also be the result of greater mean curvature estimates at lower osmotic potential.

The relationship between the logarithmic index (logarithms of the ratio of length to the longest diameter of the convex hull) and osmotic potential revealed an interesting pattern for the different lines (Fig. 7b). In contrast to breeding lines KJ21 and B403D, that had monotonic increases in their logarithmic indices as osmotic potential decreased, indices for *cv.* Diamondbird and *cv.* Silverstar decreased over 2 MPa reaching a minimum at −2.0 and −2.40 MPa, respectively, before increasing. This indicated that the diagonal of the convex hull increased relative to the leaf strip length, implying that the leaf strip was “un-rolling” in the first instance. This is consistent with the revolute cross-section of these genotypes at full turgor (Additional file 5). To roll inwards, the leaves of these genotypes had to first flatten-out.

### Variation in length of the leaf strips

On average, the data indicated that the leaf-rolling lines KJ21 and KJ41 had longer leaf strips (data not shown). However, the analysis of variance for strip length indicated there were no differences between rolling groups (P = 0.858) but a significant effect of PEG level (P < 0.001). The rolling group × PEG level interaction was also highly significant (P < 0.001) for strip length. This indicated that across PEG levels the length of the strip was not constant, and was actually decreasing or shrinking differentially between groups: the high-roller group had a strip length decreasing more at lower leaf water potential than the low-roller group. It is also worth mentioning that leaf lengths for individual lines were statistically different (P < 0.05) (data not shown).

### Scaled indices

Comparisons of “scaled” mean-curvature, i.e. mean curvature divided by estimated leaf strip length, to mean curvature “unscaled” did not indicate a different pattern between genotypes. Conclusions from the analysis of variance on scaled mean-curvature were identical to conclusions for unscaled mean curvature, i.e. highly statistically significant effects of rolling group, PEG concentration (P < 0.001), and of their interaction*, i.e.* rolling group × PEG concentration (P < 0.001). Mean curvature was thus unaffected by scaling, i.e. the length of the leaf strip was not playing a major role in explaining variation in leaf rolling.

The analysis of variance for the logarithm of the length divided by the greatest diameter of the convex hull with trace(S) = 10 showed the expected effects including statistically significant differences between the lines and the different degrees of leaf dehydration. The convex hull was unaffected by the smoothing and interpolation but the estimation of the length of the strip was affected (data not shown). No significant differences (P > 0.05) were identified for genotypes within a group, indicating that behaviours within a rolling group were similar. At high osmotic potentials, the rolling group comprising breeding lines KJ21 and KJ41 had noticeably higher ratios than the non-rolling group, the differences becoming more pronounced at intermediate and lower osmotic potentials.

## Discussion

Unlike the traditional methods for quantifying leaf rolling, i.e. leaf-rolling scores and rolling indices, quantifying leaf rolling using the approach detailed here with PEG solutions allowed evaluation of diverse genotypes under essentially identical conditions. Measuring leaf rolling using this protocol was independent of external confounding factors such as variation in vapour-pressure deficit during the day or field variation in soil water content. It was clearly demonstrated that mean curvature was very sensitive to changes in hydration level and occurred in a monotonic way over a large range of leaf water potentials. This monotonic characteristic makes mean curvature a suitable measure for assessing the ability of a genotype to roll. Monotonic increase in curvature was also reported by Moulia [7] for a single genotype of maize in characterising the mechanics of leaf rolling.

An asymmetry in transverse shapes was observed at full turgor for most lines, i.e. one side was more “rolled” than the other (Fig. 2). Interestingly, when the leaf was at the lowest osmotic potential, i.e. at −2.82 MPa, the side that was initially strongly revolute in *cv.* Silverstar did not achieve a positive concavity while the other half, which was less revolute at full turgor, did achieve a positive concavity. Equally, the side that was highly rolled in line KJ21 rolled more than the opposite side even at intermediate leaf water potential. This suggested that the transverse shape at full turgor, in particular the degree of positive or negative concavity, restricted to some extent the ability of a leaf to roll. We thus hypothesize that leaf rolling is actually mechanically impeded by the initial degree of transverse curvature at full turgor.

It is still uncertain why “shrinkage” of the strip length was more pronounced in the high-rolling group. Two explanations were hypothesised. The first was that this effect is an artefact of the data fitting. Although the recording process was similar between genotypes, the high rollers had greater curvatures around their leaf strip extremities, which the fitted spline functions tended to underestimate. Integrating $\dot{s}$ may have artificially reduced leaf strip length in the same manner that the smoothing matrix with lower traces lowered estimates of leaf strip length as a result of “over-smoothing”. The second hypothesis was that “shrinkage” is a physiological response to higher level of osmotic stress. Cells of the leaf strip in the leaf-rolling groups may have been more susceptible to loss of water and lost volume more readily than cells of the non-rolling group, resulting in differential shrinkage between these two groups.

Although mean curvature and the logarithm of the ratio of strip length to maximum diameter of the convex hull were both suitable measures of leaf rolling, mean curvature over the whole strip length was the index of “rolled-upness” that will be retained from this study as it is easier to interpret and understand than a logarithmic index. Differences between rollers and non-rollers could also be assessed at full turgor with this metric since genotypes with a high propensity for rolling had positive mean curvatures while genotypes with a lower propensity for rolling had negative curvatures. Finally, mean curvature does not need to be scaled to be meaningful and interpreted, while the logarithmic index requires the calculation of an extra parameter, which is associated with an extra standard error.

For the statistical geneticist seeking mechanisms and underlying genetic control for the leaf rolling phenotype, the method herein has the advantage of being objective and provides measures containing values that are continuously distributed. Although the method was developed to quantify leaf rolling in response to leaf dehydration, there are many other situations where leaf rolling is of interest and where this method could be applied: the assessment of damage caused by insects (e.g. wheat curl mite) [11], or potassium deficiency. The method could also be applied to other cereal crops such as sorghum, rice, maize, durum wheat and barley without having to define a different visual scale for each species.

## Conclusions

A method applying cubic smoothing splines to skeletonised images of transverse wheat leaf sections enabled objective measurements of inter-genotypic variation for hydronastic leaf rolling in wheat. Mean-curvature of the leaf cross-section was the measure selected to discriminate between genotypes, as it was straightforward to calculate and easily construed. The method has broad applicability and provides an avenue to genetically dissect the trait in cereals.

## Methods

### Plant material

Wheat (*Triticum aestivum* L.) genotypes KJ41, KJ21, B403D and cultivars Arrino, Diamondbird, Kite, Krichauff, Lang and Silverstar were sown into soil-filled wooden seedling trays (600 mm long × 300 mm wide × 100 mm deep) on 30 August 2004 at CSIRO Black Mountain (Canberra, Australia). Lines KJ41 and KJ21 are two CSIRO inbred breeding lines derived from a cross between line K648R (“roller” phenotype) and *cv.* Janz (“non-roller” phenotype). They were selected in this study due to their propensity to roll quickly with water deficit as was B403D (“roller” phenotype) while *cvs.* Silverstar and Diamondbird are usually described as “non-rolling” phenotype. The other four entries were included to randomly represent Australian cultivars without any a priori knowledge of their leaf rolling abilities, although Chara and Krichauff have been observed to roll on a few occasions (Dr N. Fettell, *pers. comm.*).

### Cultural conditions

The seedling tray contained a fertile, compost-based potting mix and was placed outdoors after sowing in order to experience “field-like” temperatures and radiation. Each entry was represented by three seeds, sown in a row-column design with three replications (9 lines × 3 reps × 3 trays). The perimeter of the tray was sown with a buffer to minimise border effect. Plants were kept well-watered and received a standard nutrient solution (modified Hoagland #2 [12]) twice while growing. Only the main stem and two tillers per plant were retained by removing regularly any newly appearing tillers, therefore limiting competition for light and water between plants. All entries reached anthesis, or Z65 [13], within a couple days of each other.

### Leaf sampling protocol and PEG treatment

At flowering, the flag leaves of individual plants were sampled by cutting the leaves just below the leaf ligule. Each leaf was then placed in a 15 mL Eppendorf tube filled with tap water and was allowed to rehydrate overnight in a constant temperature room set at 8 °C under low light conditions, so as to attain a fully turgid state. The next day each flag leaf was cut to form a 30 mm segment with the middle of the 30 mm segment being at 30 % of the maximum leaf length (Additional file 3). Preliminary work indicated that this section of the leaf was instrumental in determining the leaf rolling response of the flag leaf. Each 30 mm segment was subsequently cut into ten 3 mm strips at right angles to the central rib. The strips were then subjected to different degrees of dehydration by immersing them in solutions of PEG 3350 (Sigma^®^ Chemical). Polyethylene glycol 3350 was chosen as an osmoticum for the study as it was assumed that its high molecular weight would prevent it from entering the cells by diffusion in the time necessary to reach equilibrium. In all, seven degrees of dehydration were used: −0.06, −0.43, −1.00, −1.38, −1.76, −2.38 and −2.82 MPa. The leaf strips, randomly allocated to the different solutions, were left to equilibrate within the solutions for 4 hours in a constant temperature room, set at 20 degree Celsius, in order to reach thermal and osmotic equilibrium. Strips were placed in a glass Petri dish and covered with tap water or solutions of PEG. Some leaves were left for 3 days in the solutions and did not show further rolling.

### Osmotic potentials of Polyethylene glycol 3350 solutions (Π_PEG_): calibration

For calibration purposes, ten solutions of PEG 3350 were prepared by dissolving from 5–55 g of PEG in 100 g of milli-Q Water. A shaker (Bioline) set up at 50 °C and 90 rpm was used to homogenise the PEG solutions for two and a half hours. The solutions were then left to equilibrate overnight in a 20 °C constant temperature room before measuring their osmotic potential. One extra solution consisting of pure milli-Q water was also included in the calibration. High concentrations of PEG, i.e. 0.50 and 0.55 g.g^−1^, were not used for assessing changes in transverse shapes of the leaf strips because it was considered that the viscosity of the solution at those concentrations could mechanically impede the rolling of the strips.

Osmotic potentials of the ten PEG solutions were measured using custom-built thermocouple psychrometers. Strips (30 × 11 mm) of filter papers (Whatman #2) lining the walls of the psychrometric chambers were soaked with 100 µL of the different PEG solutions. The psychrometric chambers were sealed and left in a constant temperature room, set at 20 °C, for 4 hours to reach thermal and water-vapour equilibrium. Osmotic potentials of the solutions were determined using a dew point microvolt meter (Wescor HR33T, Inc Utah, USA) operated in the dew point mode.

The relationship between PEG 3350 and osmotic potential at 20 °C is plotted in Additional file 4. The relationship was fitted by a second-order polynomial equation and described by the following: Π_PEG_ = −11.517[PEG]^2^−1.0508[PEG]−0.0342 with Π_PEG_ in MPa and [PEG] in g.g^−1^ of milli-Q water.

### Micro-photographs of leaf transverse shapes

**Fig. 8:**
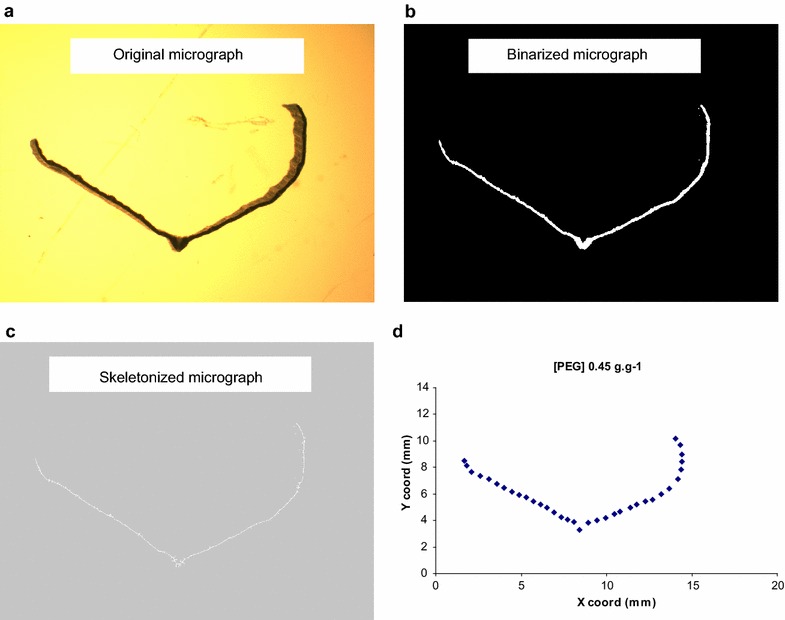
Sequence for processing the image: **a** original cross-section, **b** binarised cross-section, **c** skeletonised cross-section and **d** data points coordinates in Microsoft Excel

A transverse digital image of each leaf strip after equilibration in the PEG solution was taken using a digital camera (Colorview II, Olympus) mounted on a dissecting microscope (Leitz M8 dissector). Each leaf strip was positioned in such a way that the leaf transverse shape was facing the field of view of the dissecting microscope. The surface tension of the solution was usually enough to maintain the leaf strip on its side while the image was recorded. Each image was 178.6 mm wide × 132.5 mm high with a resolution of 2080 × 1544 pixels in a 24-bit RGB colour space. The image was acquired by setting up the camera control for acquisition in the following way: exposure time 40 ms; colour settings 2.75 for red, 1.00 for green; 1.13 for blue, 0.50 for gamma and 0.20 for saturation. This resulted in an overexposed image which gave better details of the transverse shape of the leaf (Fig. 8a). The image was then “binarised” (Fig. 8b) to isolate only the relevant information (i.e. the leaf strip) and “skeletonised” (a computer vision process which generates a thin version of the shape that is equidistant to its boundaries) (Fig. 8c). Coordinates of 17 points plus an additional point marking the midrib (18th point) on each half of the strip (left and right hand of the central rib) were then manually recorded as two vectors ***x*** and ***y***. This number of points was found to be adequate to describe the transverse leaf shape. The data were transferred to Microsoft^®^ Excel™ for plotting (Fig. 8d) and the resulting graph was visually compared to the original image.

### Statistical analysis

The lines and cultivars were divided into four groups according to the expected extent of rolling. The first group, most rolling expected, consisted of breeding lines KJ21 and KJ41, the next was *cv.* Krichauff only, B403D only, and the remaining group “non-rolling” consisted of *cvs* Arrino, Diamondbird, Kite, Lang and Silverstar. These groups are later referred to as “rolling group” in text and tables.

A Genstat program (Genstat 9, release 9.1) [14] was used to read the Excel file and for plotting the indexed data. The statistical software R (R, v 2.2.1) [15] was used to fit the convex hull and the spline functions to the data. (Program code is available on request). Smoothing splines were fitted using the R packages stats ‘smooth.spline’ [15]. The function ‘smooth.spline’ fits cubic smoothing spline to the supply data, i.e. the set of (x, y) coordinates describing the leaf transect. Mean curvature for each cross section was obtained by averaging all curvature measurements along each cross section. Calculated variables were analysed using ANOVA and mixed linear models to assess the significance of the differences between treatment, i.e. rolling group and PEG concentration. Genstat was used to fit the different models using ANOVA and REML structure with rolling group, cultivar within rolling group, and PEG concentrations considered as fixed effects and leaf side within replicate within cultivar treated as nested random effects.

## Additional files

10.1186/s13007-015-0095-1 Series of cross-sectional shapes at seven levels of leaf water potential for cv. Silverstar.
10.1186/s13007-015-0095-1 Series of cross-sectional shapes at seven levels of leaf water potential for breeding line KJ21.
10.1186/s13007-015-0095-1 Excision of strips from the leaf segment and experimental set-up schematic.
10.1186/s13007-015-0095-1 Quadratic relationship between concentration of polyethylene glycol 3350 and osmotic potential at 20 °C.
10.1186/s13007-015-0095-1 Cross-sectional shapes of the lines used in the study at full turgor.

## Abbreviations

PEG: polyethylene glycol
ANOVA: analysis of variance
REML: restricted maximum likelihood
*cv.*: cultivar
*cvs*: cultivars
