# Supplementary figures and images for: “Rolled-upness”: phenotyping leaf rolling in cereals using computer vision and functional data analysis approaches
Source: Plant Methods. 2015 Nov 14;11:52. doi: 10.1186/s13007-015-0095-1 (PMC4650205; doi:10.1186/s13007-015-0095-1)

## Slide 1
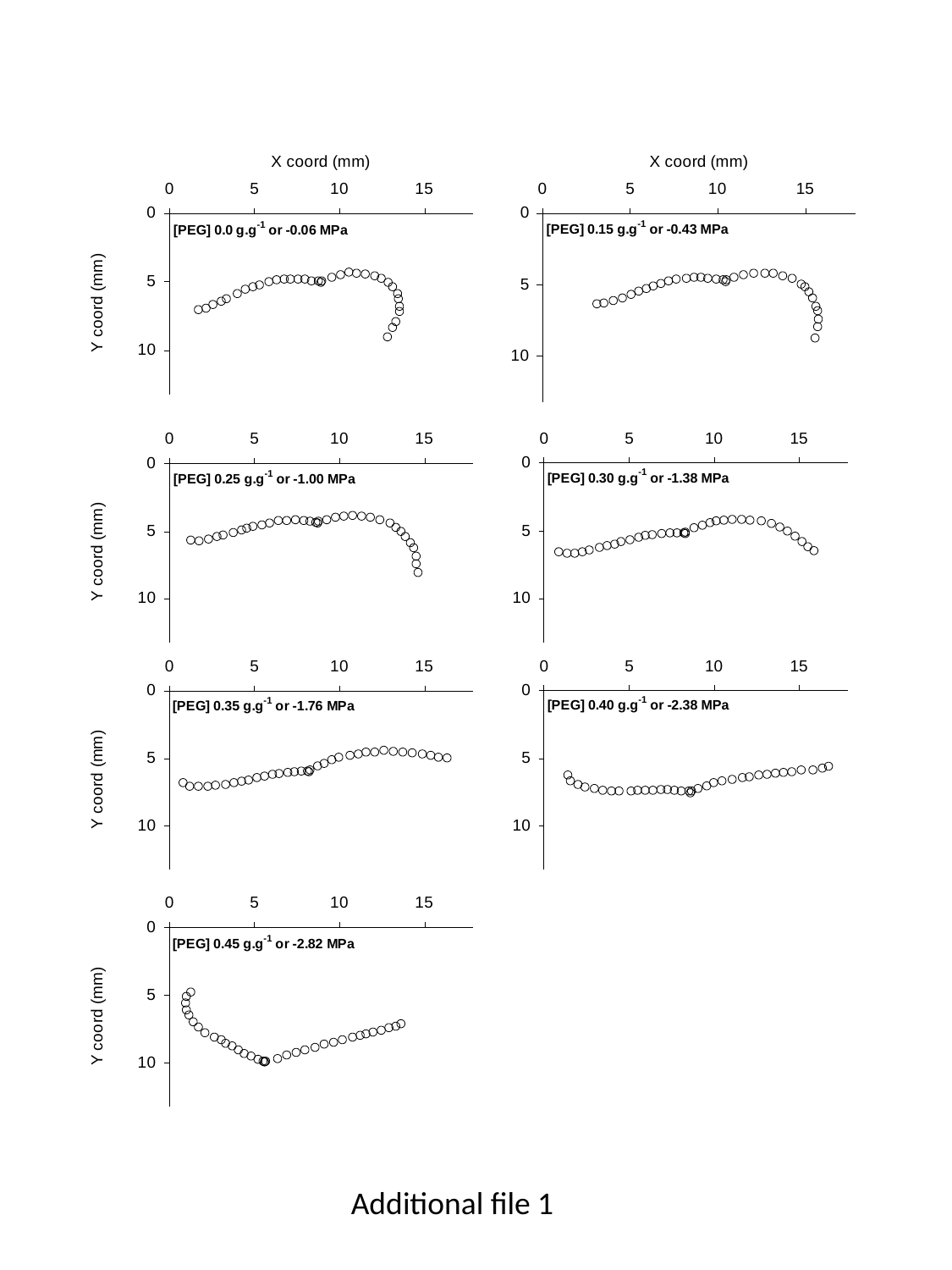

Additional file 1

Supplement: Supplementary file 1 — 10.1186/s13007-015-0095-1 Series of cross-sectional shapes at seven levels of leaf water potential for cv. Silverstar. [file 13007_2015_95_MOESM1_ESM.pptx]

## Slide 1
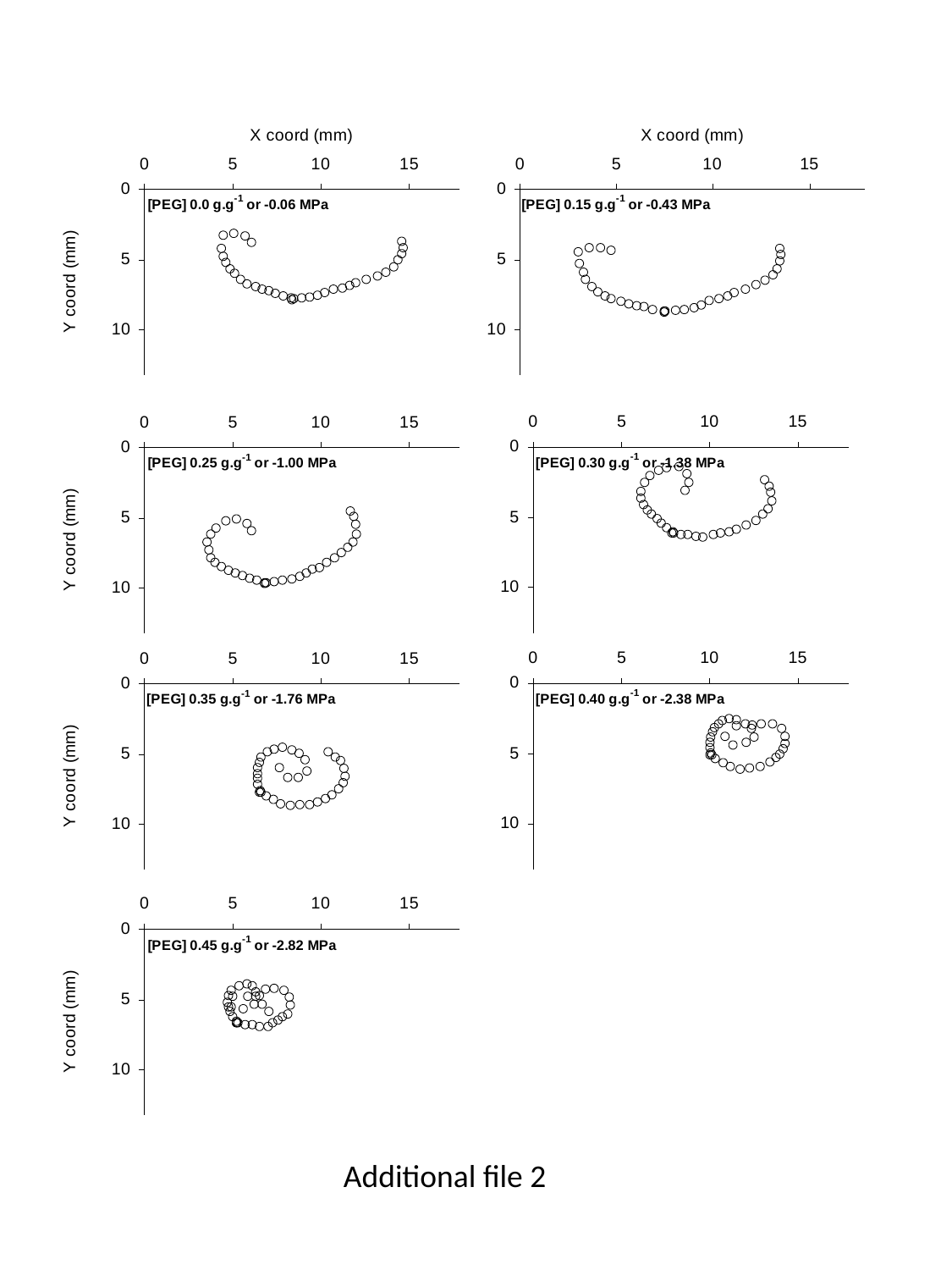

Additional file 2

Supplement: Supplementary file 2 — 10.1186/s13007-015-0095-1 Series of cross-sectional shapes at seven levels of leaf water potential for breeding line KJ21. [file 13007_2015_95_MOESM2_ESM.pptx]

## Slide 1
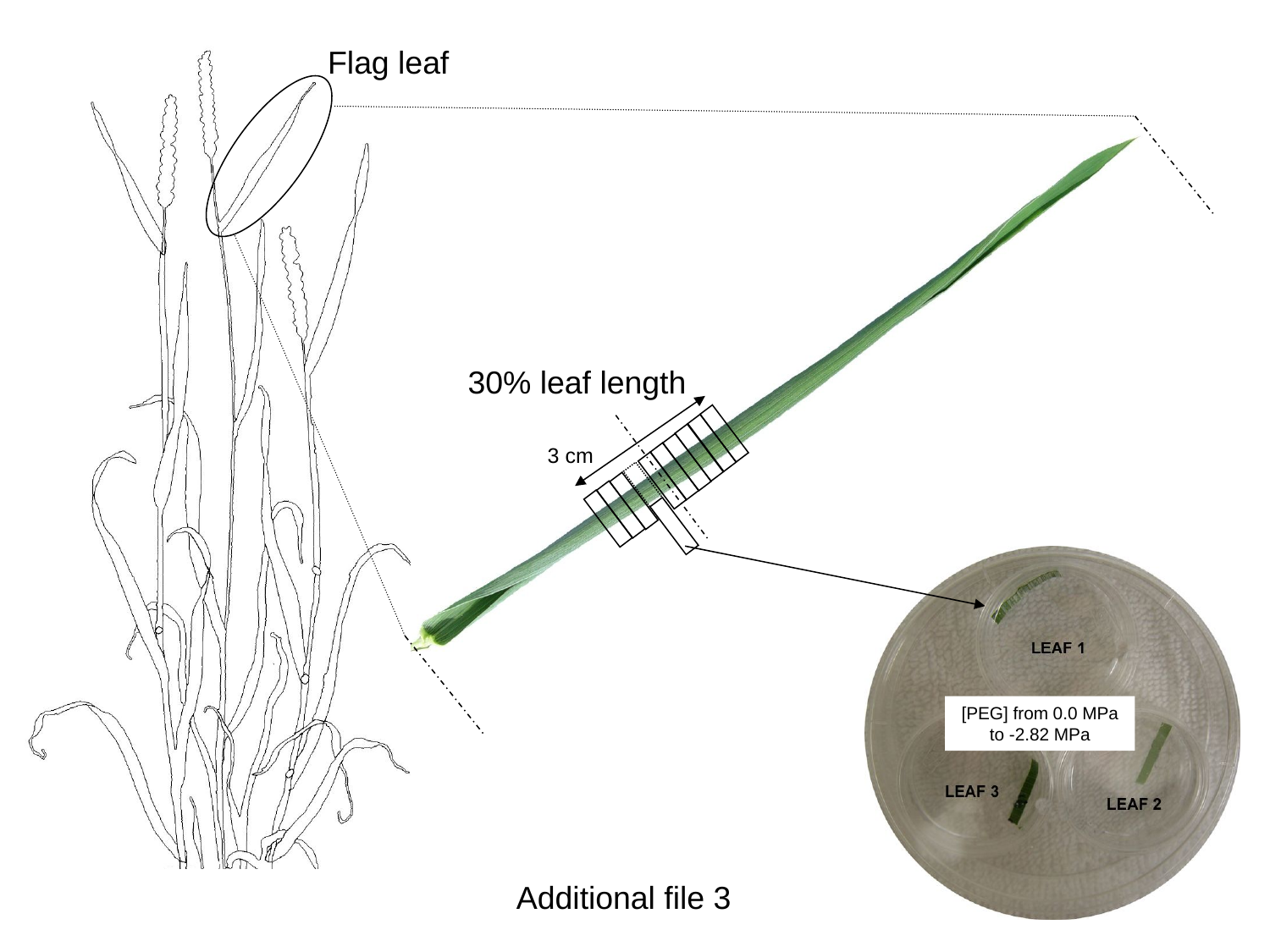

Flag leaf
30% leaf length
3 cm
[PEG] from 0.0 MPa
to -2.82 MPa
Additional file 3

Supplement: Supplementary file 3 — 10.1186/s13007-015-0095-1 Excision of strips from the leaf segment and experimental set-up schematic. [file 13007_2015_95_MOESM3_ESM.pptx]

## Slide 1
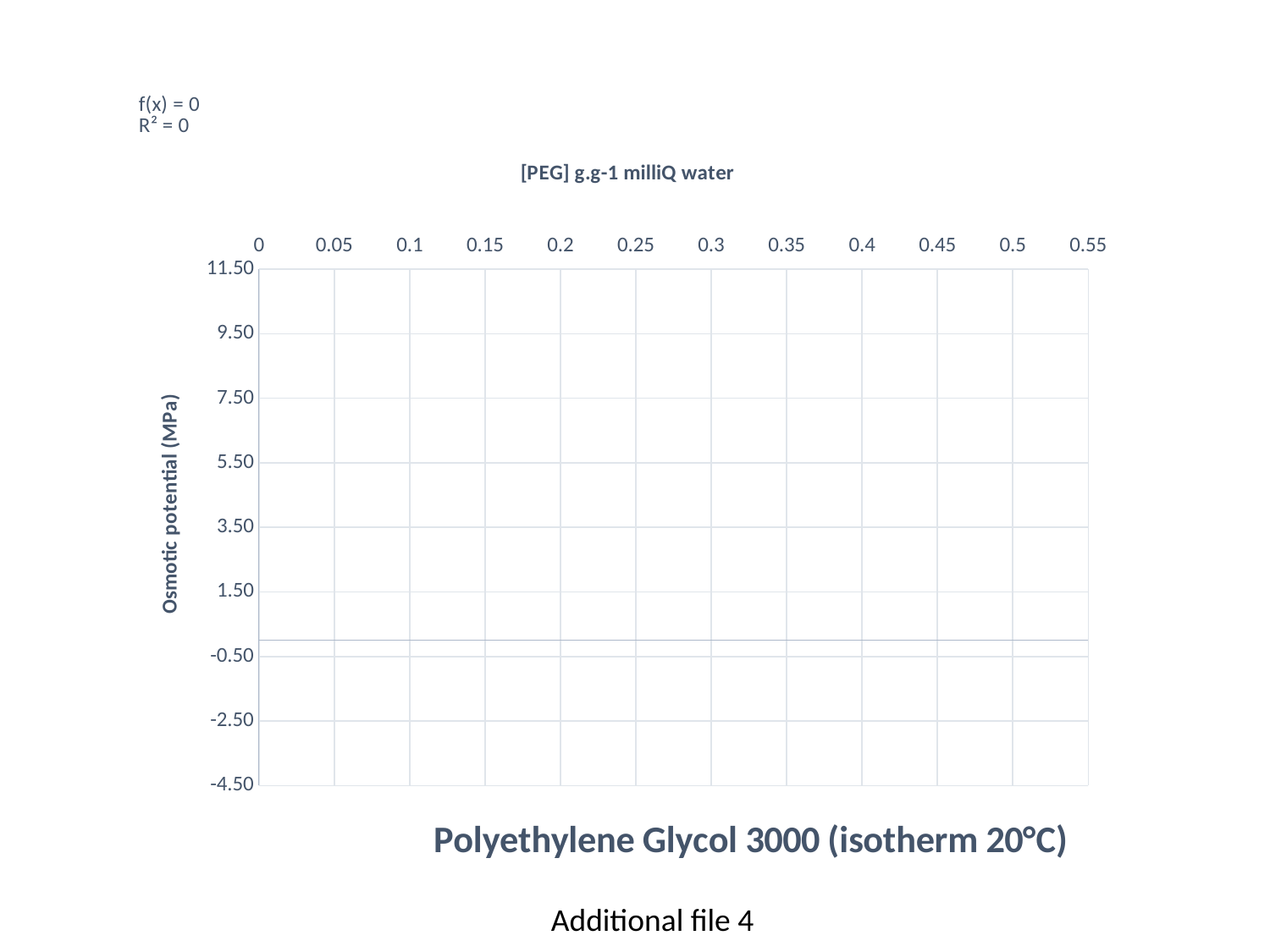

### Chart: Polyethylene Glycol 3000 (isotherm 20°C)
| Category | |
|---|---|Additional file 4

Supplement: Supplementary file 4 — 10.1186/s13007-015-0095-1 Quadratic relationship between concentration of polyethylene glycol 3350 and osmotic potential at 20 °C. [file 13007_2015_95_MOESM4_ESM.pptx]

## Slide 1
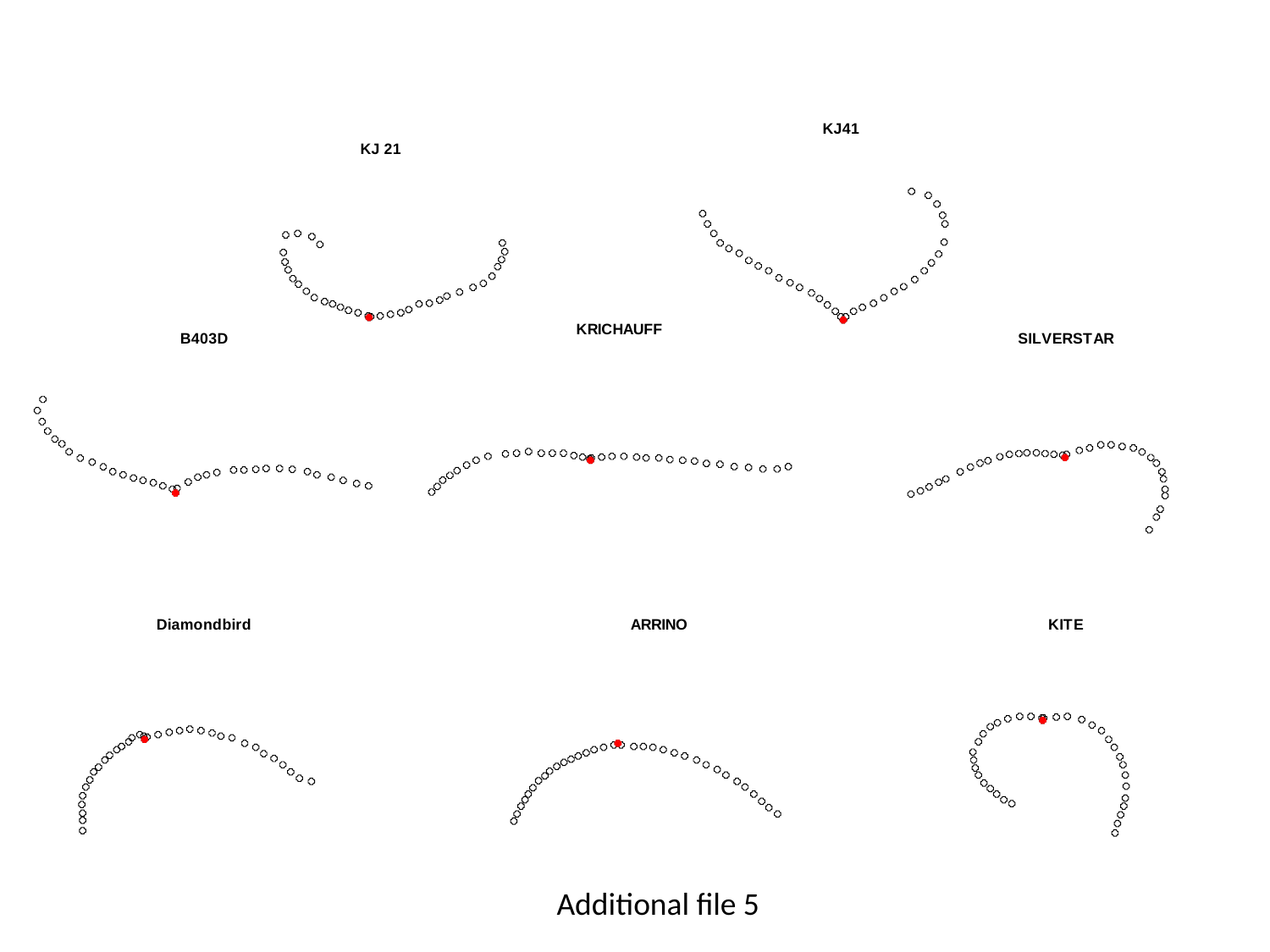

Additional file 5

Supplement: Supplementary file 5 — 10.1186/s13007-015-0095-1 Cross-sectional shapes of the lines used in the study at full turgor. [file 13007_2015_95_MOESM5_ESM.pptx]
